# Screening of cellulose-degrading bacteria and its degradation and growth-promoting applications

**DOI:** 10.1093/jimb/kuaf026

**Published:** 2025-08-29

**Authors:** Mengke Chen, Xuebin Li, Er Meng, Changjun Liu, Qinyu Li

**Affiliations:** Hunan Engineering Research Center of Lotus Deep Processing and Nutritional Health Sciences, Hunan University of Science and Technology, Xiangtan, Hunan, 411201, P. R. China; Hunan Engineering Research Center of Lotus Deep Processing and Nutritional Health Sciences, Hunan University of Science and Technology, Xiangtan, Hunan, 411201, P. R. China; Hunan Engineering Research Center of Lotus Deep Processing and Nutritional Health Sciences, Hunan University of Science and Technology, Xiangtan, Hunan, 411201, P. R. China; School of Life and Health Sciences, Hunan University of Science and Technology, Xiangtan, Hunan, 411201, P. R. China; Key Laboratory of Genetic Improvement and Multiple Utilization of Economic Crops in Hunan Province, Hunan University of Science and Technology, Xiangtan, Hunan, 411201, P. R. China; Hunan Engineering Research Center of Lotus Deep Processing and Nutritional Health Sciences, Hunan University of Science and Technology, Xiangtan, Hunan, 411201, P. R. China; School of Life and Health Sciences, Hunan University of Science and Technology, Xiangtan, Hunan, 411201, P. R. China; Key Laboratory of Genetic Improvement and Multiple Utilization of Economic Crops in Hunan Province, Hunan University of Science and Technology, Xiangtan, Hunan, 411201, P. R. China; Hunan Engineering Research Center of Lotus Deep Processing and Nutritional Health Sciences, Hunan University of Science and Technology, Xiangtan, Hunan, 411201, P. R. China; School of Life and Health Sciences, Hunan University of Science and Technology, Xiangtan, Hunan, 411201, P. R. China; Key Laboratory of Genetic Improvement and Multiple Utilization of Economic Crops in Hunan Province, Hunan University of Science and Technology, Xiangtan, Hunan, 411201, P. R. China

**Keywords:** Chaff, Cellulose-degrading bacteria, Nitrogen-fixing complex microbial agent

## Abstract

More than one billion tons of chaff waste are generated globally every year, but traditional recycling methods face the dual challenges of inefficiency and environmental risks, to solve this problem, this study innovatively achieves the dual functions of lignocellulosic synergistic degradation and plant promotion by constructing synthetic microbial communities. Firstly, a cellulose-degrading bacterium cmk-7 (*Chromobacterium violaceum*) was screened from soil based on Congo red staining method, and the maximum values of CMCase enzyme activity and FPase enzyme activity were 289.12 and 332.95 U/mL, respectively, and the culture conditions of cellulose-degrading bacteria were optimized by single factor test and response surface experiment, and its production intensity was increased by 2.43 times, respectively. Subsequently, cellulose-degrading bacteria were mixed with nitrogen-fixing bacterium Enterobacter tabaci lmy-3-2 in a 1:1 ratio to prepare a composite bacterial agent A7 to treat rice husks for potting experiments and seedling experiments. After 80 days of fermentation, the surface structure of rice husk, the soil microbial community structure was significantly reconstructed, and the ratio of carbon and nitrogen content in the soil was changed, and the plant height growth of the compound agent A7 treatment group increased by 96.5% and 193.9%, respectively, compared with the Sterile water treatment and nitrogen-fixing single bacteria treatment group, which effectively promoted the growth of buckwheat seedlings. In this study, the triple effect coupling of “solid waste degradation-soil improvement-crop growth” was successfully realized, and a mass-produced microbiome solution was provided for the agricultural circular economy, with broad application prospects.

**One-Sentence Summary**: The cellulose-degrading bacterium cmk-7 was screened and optimized to make a compound microbial agent with nitrogen-fixing bacterium lmy-3-2, which could promote chaff degradation and crop growth.

## Graphical Abstract

**Graphical Abstract:**
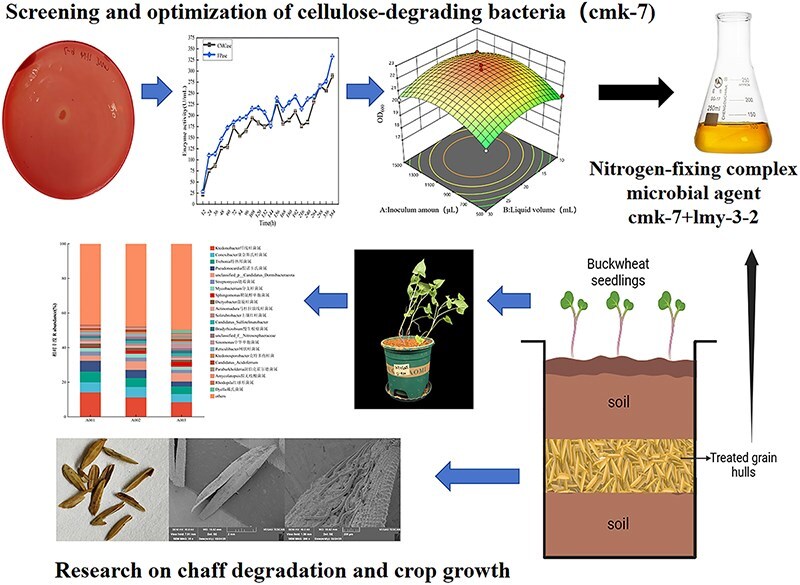

## Introduction

As one of the main sources of food for human beings, rice will produce many by-products such as rice bran, straw, husk, etc. in the process of processing into edible rice polished rice, among them, the husk, as the outermost hard protective layer of rice, accounts for 20% of the weight of rice seeds, and is the most abundant by-product in rice processing (Muthayya et al., 2014). As a valuable lignocellulosic biomass material, husk can be processed into various types of by-products, which not only enables the reuse of renewable resources such as crop waste, but also solves many ecological pollution problems (Lakshmi et al., 2008). At present, the utilization methods of rice husks at home and abroad are reflected in various fields, but the utilization rate of rice husks is still relatively low (Liu et al., 2010). The main components of husk, cellulose, hemicellulose, and lignin, are usually cross-linked with each other to form lignocellulose complexes, which are particularly strong in their three-dimensional network structure, which is the fundamental reason why husk is difficult to utilize, among which cellulose structure has high crystallinity, is insoluble in water, and is a barrier against microbial and enzyme attack (Li et al., 2020). With the global popularization of the large-scale and intensive rice processing industry, a large amount of rice husks have accumulated. therefore, solving the problem of comprehensive utilization of rice husks is an urgent task.

The degradation process of cellulose requires a critical processing step—pretreatment. Compared to high-energy physical methods, using chemical reagents such as acids, bases, and organic solvents for pretreatment is more efficient, however, this method inevitably generates intermediate toxic products, and the acid and alkali pretreatment solution has strong corrosiveness, requiring high requirements for reaction equipment and causing certain pollution to the environment, therefore, low-cost and high safety biological pretreatment is gradually replacing chemical methods and becoming a more attractive choice (Hernández-Beltrán et al., 2019). Biological pretreatment mainly relies on microorganisms and enzymes to degrade biomass in a milder manner, compared with enzyme pretreatment, microorganisms exhibit better pretreatment effects in anaerobic digestion due to their higher functional diversity and tolerance to environmental factors such as temperature and pH (Anto et al., 2020; Hernández-Beltrán et al., 2019).

There are a large number of microbial communities in nature that produce cellulase, which are generally harmless to humans and animals. Through the isolation, screening, and domestication of cellulase-producing strains, the efficiency of cellulose resource reuse can be improved. However, the complex structure, dense crystals, and stable hydrogen bonds of cellulose make it difficult to degrade, and typically require multiple cellulases to work together (Nishiyama et al., 2003). It is difficult for a single strain to produce a variety of enzymes that are highly efficient for degradation, so the degradation ability of a single strain to chaff is limited, in recent years, many studies have reported that a variety of different strains are combined to form a composite strain through enzyme complementation, and the synergistic combination can significantly improve the degradation effect. Li's team constructed a complex co-degradation system of cellulose treated by high-efficiency cellulose bacteria and mature fungi during aerobic composting of corn straw and cow dung, which can help to decompose cellulose more effectively (Li et al., 2023). Song's research found that adding microbial agents can significantly improve the composting efficiency of crop wastes, and at the same time, adding microbial agents can promote composting and reduce the adverse effects of composting products on plant germination and growth (Song, 2019). These studies indicate that the degradation efficiency of a single strain is relatively low, while composite strains constructed from multiple single strains can significantly improve enzyme activity through coordinated interactions between strains, thereby more efficiently degrading cellulose and converting it into usable monosaccharides and other carbon compounds. Therefore, the development of high-performance and robust microbial consortia represents a critical research focus. This approach aims to overcome the inherent degradation limitations of single-strain systems while enhancing their adaptability to complex environmental conditions. During the process of microbial degradation of rice husks, a large amount of nitrogen is consumed. Therefore, returning rice husks to the field causes an imbalance in soil carbon nitrogen ratio, leading to a decrease in crop yield. Nitrogen-fixing microorganisms can absorb nitrogen from the air for their own growth and reproduction, and can also secrete excess nitrogen into the soil to provide nitrogen nutrition for the growth and reproduction of other organisms (Lin et al., 2023), Compared to soil treated with a single bacterial strain, soil treated with nitrogen fixing compound agents showed an increase in phosphorus, potassium, and nitrogen content (Zhen et al., 2019), the utilization of these elements and carbon compounds varied depending on the type of microorganisms in the soil.

This experiment will use rice husks as the sole carbon source to collect soil samples containing rice husks from the field for strain enrichment. After initial screening with Congo red staining, cellulose degrading bacteria will be rescreened based on the activity of carboxymethyl cellulase (CMCase) and filter paper enzyme (FPase). Combined with morphological characteristics and 16S rDNA sequence analysis, the systematic classification status of the screened strains will be clarified; Optimize the enzyme fermentation conditions using single factor experiments and response interviews; A microbial composite preparation was prepared by combining the screened strains with nitrogen-fixing bacteria. Through a simulated pot experiment of rice husk degradation, the composition of various substances in the soil was determined, and the metagenomic information such as soil microbial community types and population density was analyzed, compare the growth status of seedlings before and after, comprehensively explore the degradation ability of composite microbial agents on rice husks and their ability to promote crop growth. This experiment screened bacterial strains that have a degrading effect on rice husks, providing a reference for rice husks returning to the field and the development of efficient rice husk biodegradable agents and cellulase preparations.

## Materials and Methods

### Materials

#### Soil samples and strains for testing

Soil samples for testing were collected from black soil containing rice husk residue in the field of Harbin Wuchang (44° 56' 25.79" N, 127° 10' 20.58" E). The tested nitrogen-fixing strain lmy-3-2 was a well-grown and environmentally adaptable *Enterobacter tabaci* strain, which was screened from the sludge at the effluent of the wastewater treatment plant.

#### Culture medium

Sodium carboxymethyl cellulose medium (CMC-Na): CMC-Na 2 g/L, Na_2_HPO_4_ 2.5 g/L, KH_2_PO_4_ 1.5 g/L, peptone 2.5 g/L, yeast extract 0.5 g/L, agar 20 g/L, PH 7.0–7.2, autoclaved at 121°C for 15 min (Sharker et al., 2023). Liquid fermentation medium: chaff powder 20 g/L, KH_2_PO_4_ 2 g/L, (NH_4_)_2_SO_4_ 2 g/L, MgSO_4_·7H_2_O 0.5 g/L, CaCl_2_ 0.38 g/L, urea 0.5 g/L, CoCl_2_·6H_2_O 5.5 mg/L, FeSO_4_·7H_2_O 7.5 mg/L, MnSO_4_·H_2_O 2.5 mg/L, ZnSO_4_·7H_2_O 3.56 mg/L, sterilized at 121°C for 20 min (Shi et al., 2023). Nutritional medium (NA): beef paste 3 g/L, Peptone 10 g/L, NaCl 5 g/L, sterilized at 121°C for 20 min. Peptone yeast sucrose medium (YSP): peptone 10 g/L, yeast powder 5 g/L, sucrose 20 g/L, sterilized at 115°C for 30 min (Shi et al., 2023). Beef paste yeast glucose medium (NYBD): beef extract 8 g/L, yeast extract 5 g/L, glucose 10 g/L, sterilized at 115°C for 30 min (Shi et al., 2023). Bacterial basal medium (CM): glucose 5 g/L, (NH_4_)_2_SO_4_ 2 g/L, sodium citrate 1 g/L, MgSO_4_·7 H_2_O 2 g/L, K_2_HPO_4_ 4 g/L, KH_2_PO_4_ 6 g/L, sterilized at 121°C for 20 min. Peptone yeast culture medium (LB): peptone 10 g/L, yeast powder 5 g/L, sodium chloride 10 g/L, sterilized at 121°C for 20 min. Bacterial seed culture medium (XJ): beef extract 3 g/L, peptone 10 g/L, KH_2_PO_4_ 1 g/L, sodium chloride 5 g/L, sterilized at 121°C for 20 min. Liquid culture medium (YT): sodium carboxymethyl cellulose (CMC-Na) 20 g/L, (NH_4_)_2_SO_4_ 2 g/L, KH_2_PO_4_ 2 g/L, FeSO_4_·7H_2_0, 0.01 g/L, CaCl_2_ 0.1 g/L, sodium chloride 5 g/L, sterilized at 121°C for 20 min (Zhang et al., 2023).

### Screening of Cellulose-Degrading Bacteria

#### Initial screening

The enrichment solution containing cellulose-degrading bacteria was diluted gradually, evenly coated on the Congo red-carboxymethyl sodium cellulose medium plate, and incubated at 37°C for 24–36 hr. After the colonies have grown, strains with different shapes and colors and transparent circles of colonies are picked with inoculation loops and inoculated into stained CMC-Na medium plates and purified by repeated striing (Khosravi et al., 2021).

#### Determination of cellulase activity

The purified strain was inoculated into liquid fermentation medium, and cultured with shaking at 37°C and 220 r/min. Samples were collected every 12 hr, centrifuged at 4°C and 4000 r/min for 20 min, take the supernatant as the crude enzyme and use DNS method to determine the activity of CMCase and FPase. Take 500 µL 1% CMC-Na solution into two test tubes (Liu, 2019)/take 500 µL 1% citric acid buffer solution into two test tubes with 25 mg starch free filter paper (Shi et al., 2018), add 250 µL of crude enzyme solution to one of the test tubes, and add 250 µL of inactivated enzyme solution to the other tube, after a 50°C water bath for 0.5 hr, add 200 µL of DNS to the sample and heat it in a 100°C water bath environment for 5 min, then quickly cool the sample to room temperature and add sterile water until the total volume reaches 2.5 mL. After completing the above steps, it is necessary to measure the absorbance value at a wavelength of 540 nm, repeat the operation three times for each sample, and finally calculate the activity level of CMCase and FPase enzyme according to the glucose standard curve. The enzyme activity unit is expressed in U/mL. The amount of enzyme required to catalyze substrate hydrolysis and release 1 µg of glucose per minute for 1 mL of enzyme solution is one enzyme activity unit, calculated using the formula (Liu, 2019):


\begin{eqnarray*}
{\mathrm{Enzyme\ activity\ }}\left( {{\mathrm{U}}/{\mathrm{mL}}} \right) = \frac{{X \times N \times \ 1000}}{{A \times T}}
\end{eqnarray*}

In the formula: U/mL—enzyme activity unit;

X—Glucose content obtained from the standard curve, mg/mL;

N—dilution ratio of enzyme solution;

A—The amount of enzyme solution added, mL;

T—enzymatic hydrolysis reaction time, min.

### Strain Identification

#### Morphological and physiological and biochemical identification

The colony morphology of the strain was observed after plate scribing culture, and a single colony smear was selected for Gram staining, and the morphology of the strain was observed and recorded using a light microscope (Khosravi et al., 2021). The bacterial solution was centrifuged at 8000 rpm for 4 min, the supernatant was removed to obtain the cell precipitate, and the 2.5% glutaraldehyde was fixed and then left at 4°C overnight for electron microscopy scanning.

#### 16S rDNA sequence analysis

The amplification of 16S rDNA sequence was carried out by colony PCR, the tested strains were cultured for 24 h, and a few colonies were selected as PCR templates. Bacterial universal primers 27F(5'-AGAGTTTGATCCTGGCTCAG-3') and 149R(5'-GGTTACCTTGTTAGACTT-3') were selected for PCR amplification. Twenty microliter of reaction system: 0.5 µL of upstream and downstream primers, 0.2 µL of Taq enzyme, 1 uL of Deoxyribonucleoside triphosphate (dNTP) (2 mM) and buffer (10×) and 15.8 µL of sterile water. The reaction conditions are: predenaturing at 95°C for 5 min; 30 cycles at 95°C for 30 s, 55°C for 45 s, and 72°C for 60 s; 72°C for 10 min. PCR products were detected by 1.0% agarose gel electrophoresis, and the products were sent to a biological company for sequencing. After the sequencing results of 16S rRNA gene of the strain were obtained, the homology was compared in NCBI database (Troussellier et al., 2005; Yakimov et al., 2006). Sequences with high similarity were obtained from GenBank and other gene databases, and the phylogenetic tree was constructed by using MEGA 11.0 software, and the genus and species attribution of the strains were analyzed.

### Optimization of Culture Conditions

#### Single factor experiment

According to the references (Biswas et al., 2024; Zhang et al., 2024), The culture conditions (culture medium, nitrogen source, inoculation amount, liquid volume, pH, rotation speed, temperature, and incubation time) of the strain were optimized, and the OD_600_ value of bacteria was used as the optimization index, and each group was repeated three times.

#### Response surface experiment

According to the results of the experimental ANOVA after the optimization of the single factor test, the optimal fermentation culture conditions were used as the central point, and the Box-Benhnken model in the Design Expert 13 software was used to design the three-factor three-level test, and the three variables with the most significant impact on the growth of the strain were further optimized with OD_600_ as the response value (Miao et al., 2015; Liu et al., 2025).

### Pot Simulation Experiment of Chaff Degradation

#### Pot experiment

Prepare the seed solution of nitrogen fixing bacterium lmy3-2 without antagonistic reaction with cmk-7 strain, mix it with the seed solution of cmk-7 strain prepared under optimized culture conditions in a 1:1 ratio to prepare composite agent A7, and conduct outdoor potted simulation experiments. The experimental method is referenced in reference (Zhang et al., 2018) and improved accordingly, each pot contains 2 kg of soil sample, with 6 g of husks treated with different microbial agents buried in the soil. Select husk bacterial agent for treatment (fresh rice husk was completely immersed in the compound bacteria solution to ensure the adhesion of bacteria), and three treatments, single treatment of nitrogen-fixing bacteria lmy-3-2, sterile water treatment (blank control) and A7 treatment of compound bacteria were set up. The experiment was carried out from October to December, and the outdoor environment was 20°C–10°C for 80 days.

#### Determination of soil reducing sugar and ammonia nitrogen content

Take husks from potted plants on the 10th, 20th, 30th, 40th, 50th, 65th, and 80th days, the surface of the husk was cleaned with 10 mL of sterile water, centrifuged at 4000 rpm for 20 min, and the supernatant was taken to determine the content of reducing sugar (Liu, 2019) and ammonia nitrogen in the soil (Hu et al., 2019).

#### Scanning electron microscope (SEM) analysis of the surface microstructure of the chaff.

After 80 days of fermentation, take out the residual husk material after fermentation, Rinse the sample with 0.1 M potassium phosphate buffer (pH = 8), filter through a 100 target sieve, and dry to constant weight, In addition, take the nonfermented husk dried sample and the fermented husk dried sample for scanning electron microscope (SEM) scanning.

### Potted Seedling Experiments

After 20 days of rice husk fermentation, buckwheat seedlings were transplanted, the growth of the seedlings before transplanting was recorded, and the growth of the seedlings was recorded regularly, and after 30 days of planting, the seedlings were removed, and the growth changes were recorded by photographs, and the remaining husk continued to ferment for 20 days (Cao et al., 2011; Zhang et al., 2024).

#### Soil microbial metagenome sequencing

Potted soil treated with compound strain A7(cmk-7 + lmy-3-2) and pot soil treated with Sterile water treatment group and pot soil without chaff fermentation were taken to remove impurities such as sand, chaff and branches, and then sent to Shanghai Meiji Biomedical Technology Co., Ltd. for metagenomics sequencing (Deng et al., 2021; Liu et al., 2025).

### Data Analysis

WPS Office, SPSS 27.0, Origin 2022, Design-expert 13 and other software were used for data analysis and chart making, the neighbor-joining method in MEGA 11.0 software was used to construct the phylogenetic tree of strains, and Meiji Cloud (https://www.majorbio.com) was used for metagenomic data analysis.

## Results and Analysis

### Screening Results of Cellulose Degrading Bacteria

**Fig. 1.**
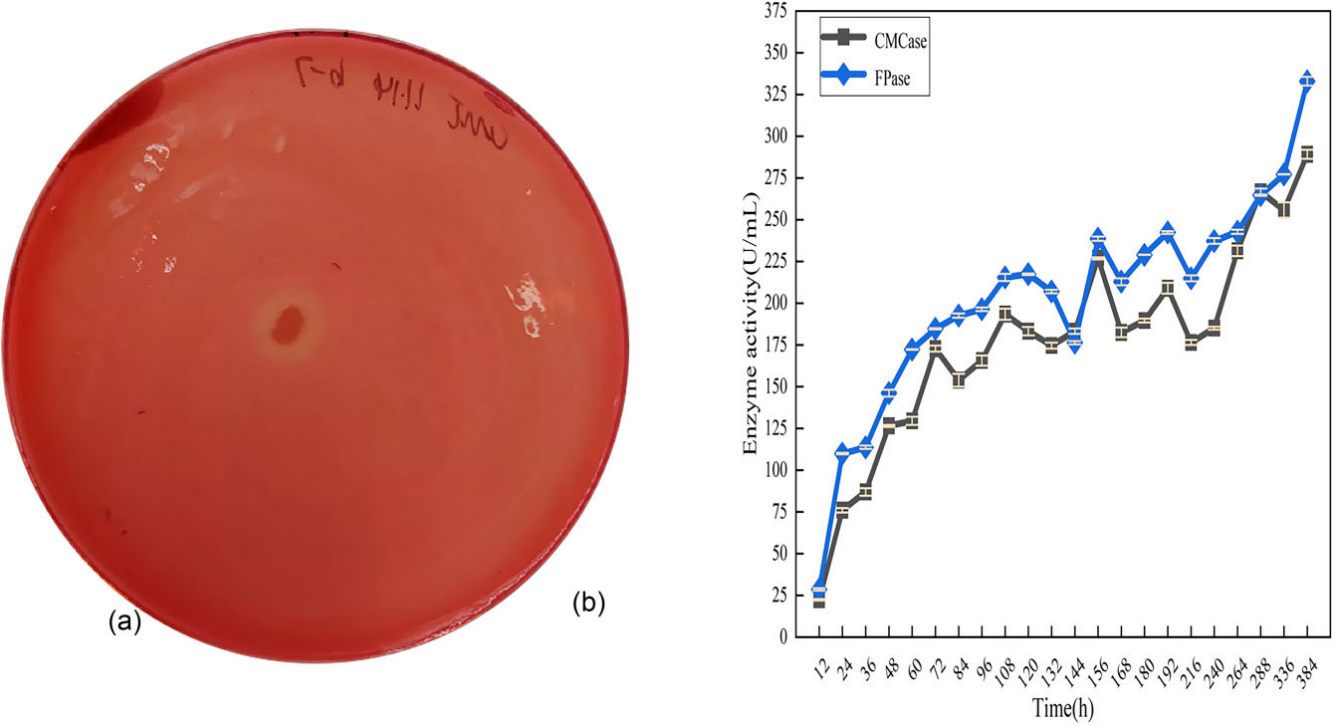
Congo red staining test results and enzyme activity assay results. A: 24 hr Congo red staining results, B: Changes in the ability of cellulose degrading strain cmk-7 to produce CMCase and FPase over time, with the size of the error bar representing significant differences (*p* < .05).

The preliminary screening results are shown in Fig. 1A: eight strains were screened through Congo red staining, among which strain cmk-7 produced significantly larger decolorization circles than the other strains, indicating that this strain has stronger potential for cellulose degradation than the other strains. Therefore, further testing was conducted on the CMCase and FPase activities of the selected cellulose-degrading bacterial strain cmk-7, as shown in Fig. 1B, the CMCase and FPase activity of cmk-7 were in a rising state before 144 hr, then in a tortuous state after 144 hr, and increased to 289.12 U/mL and 332.95 U/mL at 384 hr, respectively, which indicated that strain cmk-7 had potential CMCase and FPase activity.

### Identification Results of Strains

#### Morphological, physiological, and biochemical identification results

**Fig. 2.**
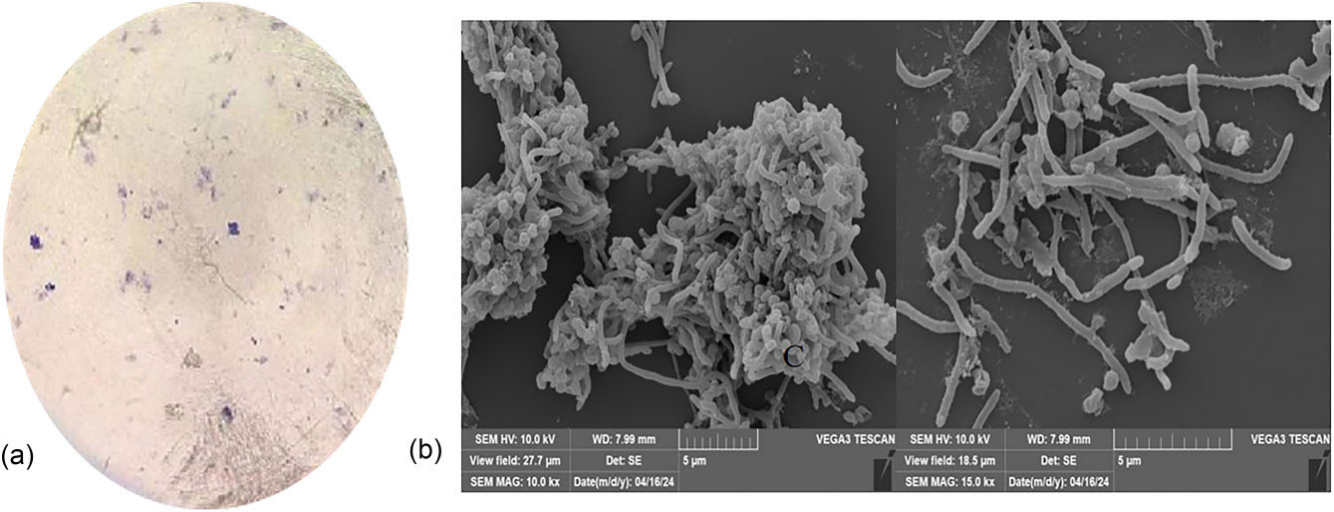
Morphological identification results of bacterial strains. A: Gram stained bacterial morphology of strain cmk-7, B: bacterial morphology of strain cmk-7 under electron microscopy scanning at 27.7 µM and 18.5 µM fields, respectively.

The cell of strain cmk-7 is nearly round and convex, and its surface is moist and shiny, but it is also opaque. It is a Gram-negative rod-shaped bacterium with blunt ends and no spore production (Fig. 2A). According to the identification results of scanning electron microscope, the following conclusions can be drawn: cmk-7 is long and dry, with a smooth surface and a sticky and entangled state, with a length of 5 µM or more and a width of 0.5–1 µM (Fig. 2B).

#### Phylogenetic tree construction and analysis

**Fig. 3.**
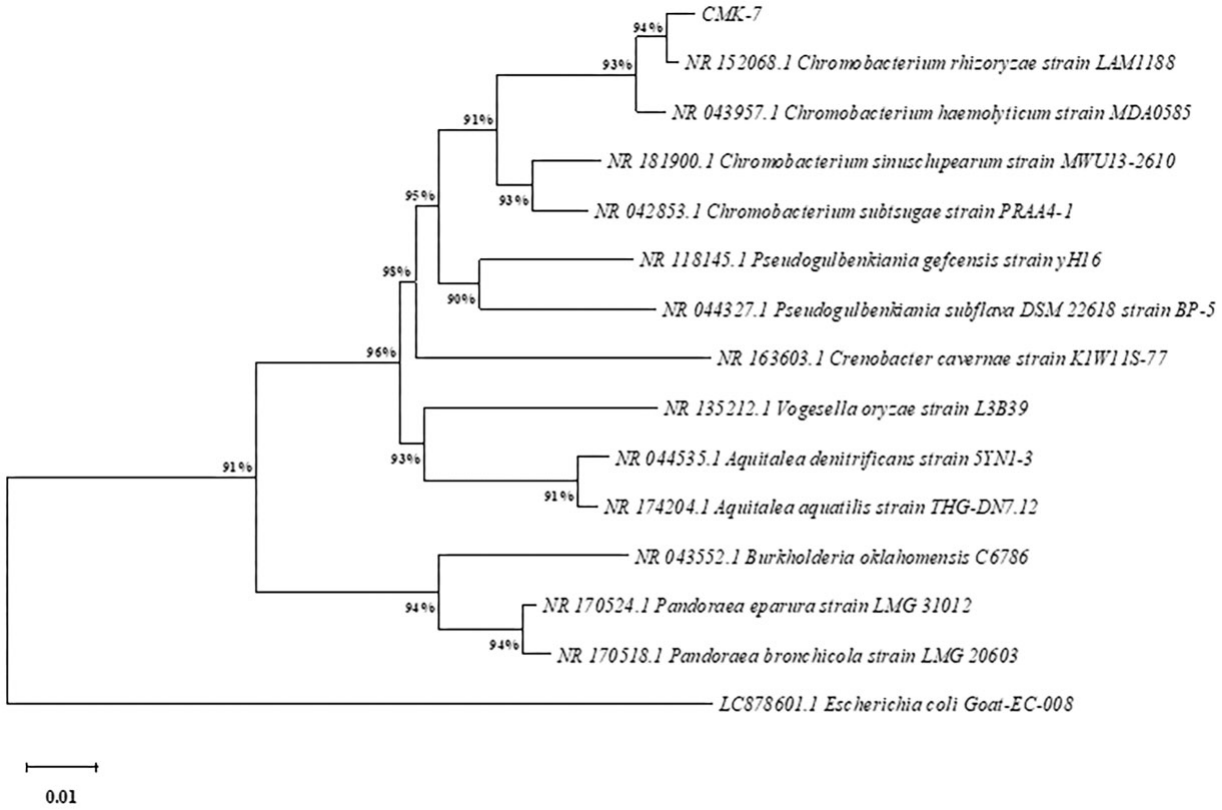
Phylogenetic tree of the strain based on the 16S rRNA gene sequence. Only >90% of the boot values are shown at the branches, and 0.01 represents the genetic distance between the two nucleosides.

The 16S rRNA sequence of strain cmk-7 was compared and analyzed by NCBI Blast, and the gene sequence with the highest similarity to the alignment sequence was selected, and the phylogenetic tree was constructed by N-J method. As shown in Fig. 3, cmk-7 is in the same branch as *NR_152068.1 Chromobacterium rhizoryzae*, with a self-expansion support rate of 94%. This indicates that cmk-7 belongs to the *Chromobacter sp*. genus and is closely related to *Chromobacterium rhizoryzae*. Therefore, it is preliminarily determined that cmk-7 belongs to the *Chromobacterium rhizoryzae* species.

### Optimization Results of Culture Conditions

#### Single factor experimental results

**Fig. 4.**
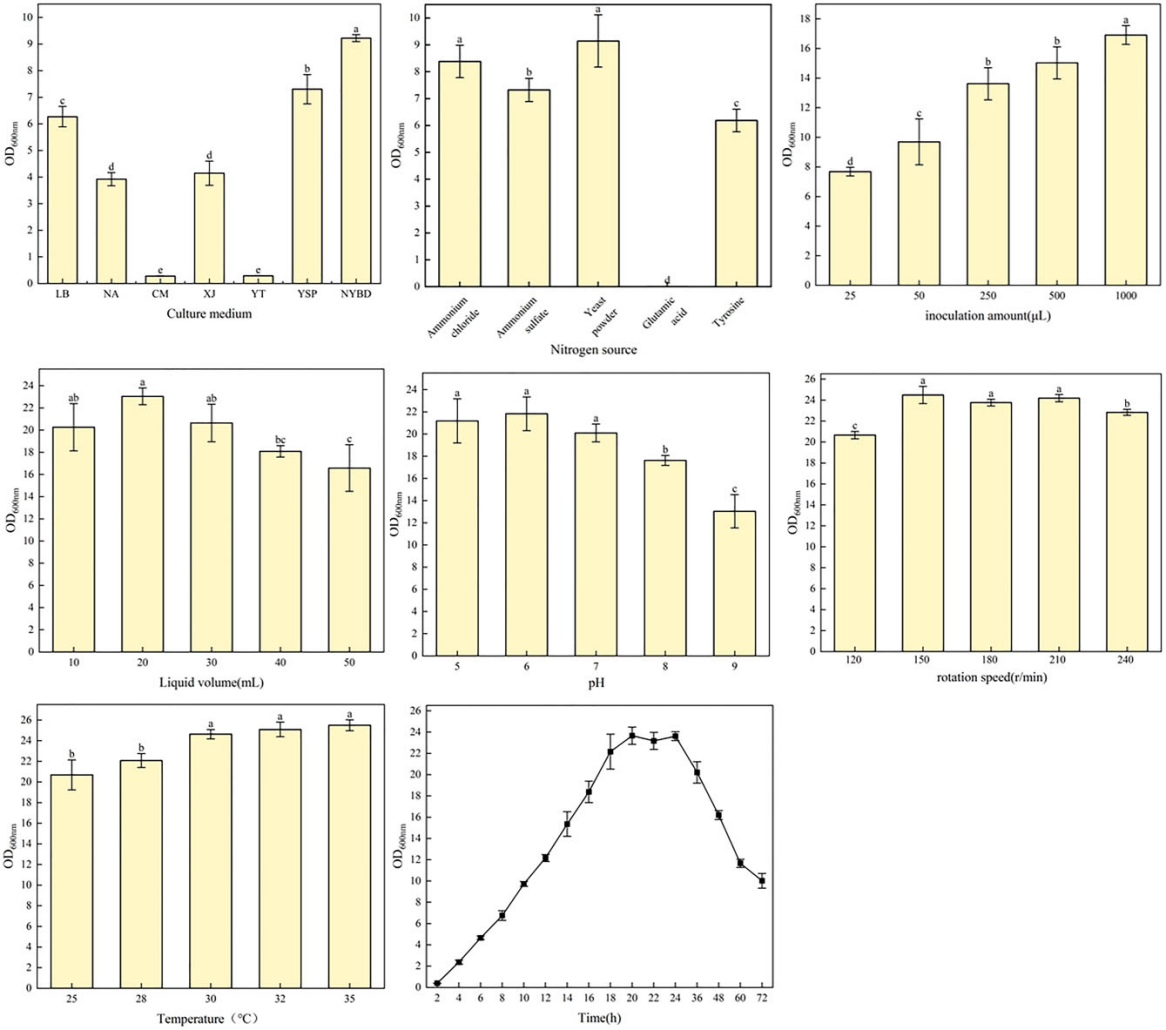
The effect of single factor (culture medium, nitrogen source, inoculation amount, liquid volume, initial pH, rotation speed, temperature, fermentation duration, etc.) culture conditions on the growth of strain cmk-7. The size of the error bar represents significant differences (*p* < .05).

By studying the effects of different culture conditions (culture medium, nitrogen source, inoculation amount, liquid volume, initial pH, rotation speed, temperature, and incubation time) on the OD_600_ of strain cmk-7, the analysis results are shown in Fig. 4. Based on the comprehensive analysis of these results, the optimum fermentation conditions of strain cmk-7 were determined: culture medium: NYBD culture, nitrogen source: yeast powder, inoculation amount: 1000 µL, liquid volume: 20 mL, pH:6, rotation speed: 150 r/min, temperature: 35°C, incubation time: 20 hr.

#### Experimental results of response surface

According to the results of ANOVA of the one-factor experiment, the three factors that had the most significant effect on the bacterial concentration of strain cmk-7 were selected as independent variables, namely inoculation amount (A), liquid volume (B), and pH (C), with OD_600_ was used as the response value, Table 1 shows the range and central point values of the three independent variable. For other factors, the medium was set to NYBD medium, the nitrogen source was set to yeast powder, the rotation speed was set to 150 r/min, the temperature was set to 35°C, and the incubation time was set to 20 hr.

**Table 1.** Response Surface Test Results (cmk-7)

	A	B	C	
Serial number	Inoculum amount (uL)	Liquid volume (mL)	pH	OD_600_
1	500	10	6	20.31
2	1500	10	6	21.02
3	500	30	6	19.94
4	1500	30	6	20.34
5	500	20	5.5	17.59
6	1500	20	5.5	18.32
7	500	20	6.5	20.42
8	1500	20	6.5	21.34
9	1000	10	5.5	18.54
10	1000	30	5.5	17.89
11	1000	10	6.5	20.52
12	1000	30	6.5	21.54
13	1000	20	6	22.14
14	1000	20	6	22.88
15	1000	20	6	22.15
16	1000	20	6	22.53
17	1000	20	6	22.4

Seventeen groups of experiments were designed by using Box-Benhnken center in Design-Expert 13 software, and the experimental results are shown in Table 1. The quadratic regression equation prediction model obtained by fitting the experimental data with multiple regression equations is as follows:

Y(OD_600_ = 22.42 + 0.345A–0.085B + 1.44C–0.0775AB + 0.0475AC + 0.4175–1.11A^2^–0.9062B^2^–1.89C^2^), the variance analysis and significance test of the obtained regression equation prediction model are carried out. As shown in Table 2, it can be seen that the *p* of the model is less than .0001, the regression model is extremely significant, and the mismatch term of the model is *p* = .4634 > .05, which is not significant, and the determination coefficient of the model is R^2^ = 98.51%, which shows that the prediction model has high fitting and can well predict and analyze the optimal fermentation conditions of strain culture.

**Table 2.** Analysis of Variance of Regression Model (cmk-7)

Source	Sum of squares	df	Mean square	F value	*p* value	Significance
Model	44.34	9	4.93	51.48	<.0001	Significant
A-inoculum amount	0.9522	1	0.9522	9.95	.0161	*
B-liquid volume	0.0578	1	0.0578	0.6039	.4625	Not significant
C-pH	16.47	1	16.47	172.13	<.0001	**
AB	0.0240	1	0.0240	0.2510	.6317	Not significant
AC	0.0090	1	0.0090	0.0943	.7677	Not significant
BC	0.6972	1	0.6972	7.28	.0307	*
A²	5.20	1	5.20	54.33	.0002	**
B²	3.46	1	3.46	36.13	.0005	**
C²	15.06	1	15.06	157.36	<.0001	**
Residual	0.6700	7	0.0957	-	-	-
Lack of fit	0.2946	3	0.0982	1.05	.4634	Not significant
Pure error	0.3754	4	0.0938	-	-	-
Cor total	45.01	16	-	-	-	-
R^2^	0.9851	-	-	-	-	-

Note: ** was the most significant difference (*p* < .01); *Significant difference (*p* < .05).

The contour plot and response surface plot of the influence of the interaction between various factors on the OD_600_ of the strain were made by using Design-Expert 13 software.

**Fig. 5.**
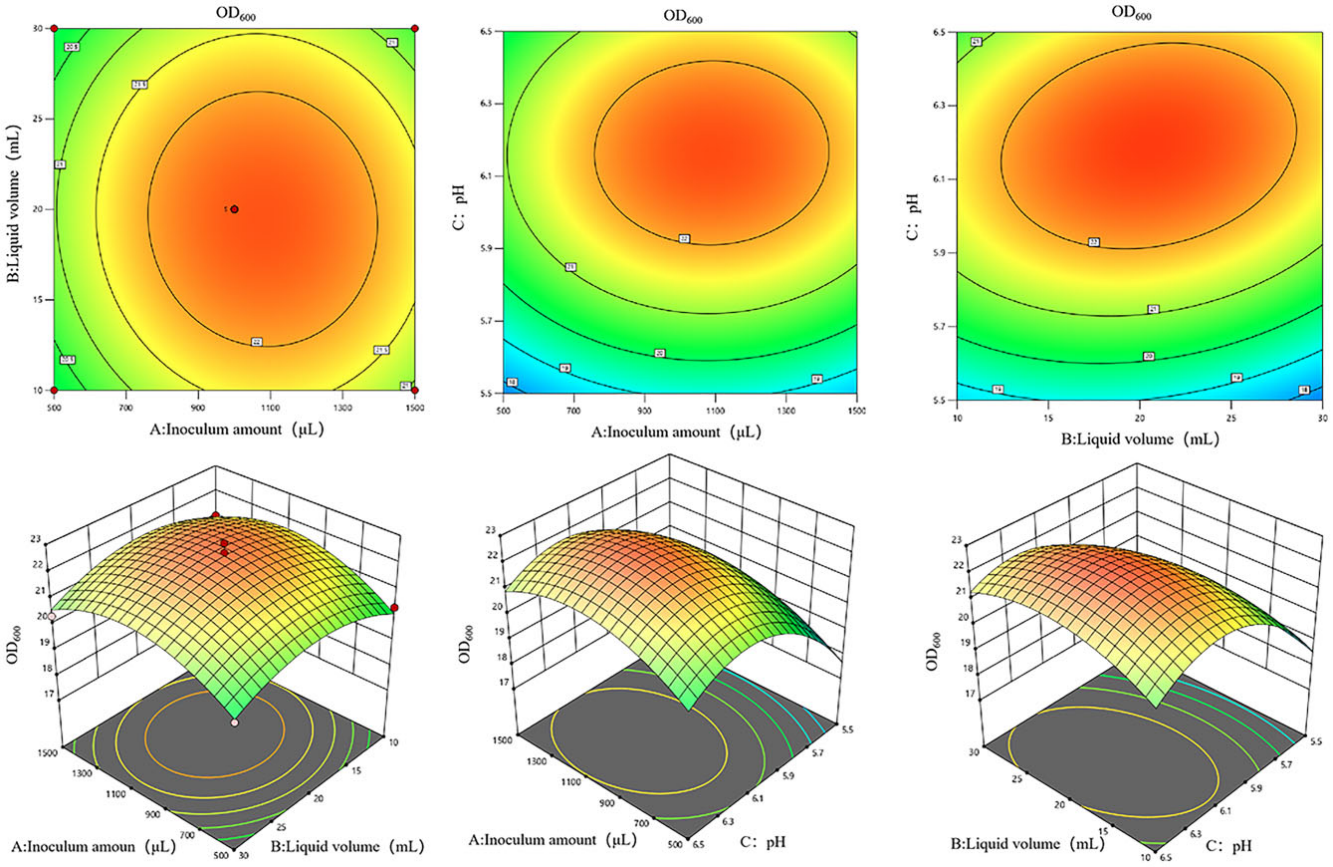
Contour diagram (top) and three-dimensional diagram (bottom) of the influence of inoculation amount, liquid volume, and pH on strain (cmk-7) OD_600_. A: Contour map and response surface map of the interaction between inoculation amount and liquid volume; B: contour map and response surface map of the interaction between inoculation amount and pH; C: contour map and response surface map of interaction between pH and liquid volume.

As can be seen from Fig. 5, the interaction between liquid volume (B) and pH (C) had the most significant effect on cmk-7 OD_600_, while the interaction between inoculation amount (A) and pH (C), inoculation amount (A) and liquid volume (B) had no significant effect on cmk-7 OD_600_. The optimal culture conditions for the strain cmk-7 were: inoculation amount: 1500 µL, liquid volume: 10 mL and pH: 6, and the maximum OD_600_ predicted value was 20.91. Under these conditions, a verification experiment was conducted, and the actual value of OD_600_ was measured to be 21.49, compared with the OD_600_ obtained under LB culture conditions before optimization, it increased by 2.43 times.The difference between the actual value and the predicted value is very small, indicating that the prediction model has a high degree of fit, accuracy, and reliability.

### Simulation Results of Chaff Degradation Potted Plants

#### Determination results of soil reducing sugar and ammonia nitrogen content

**Fig. 6.**
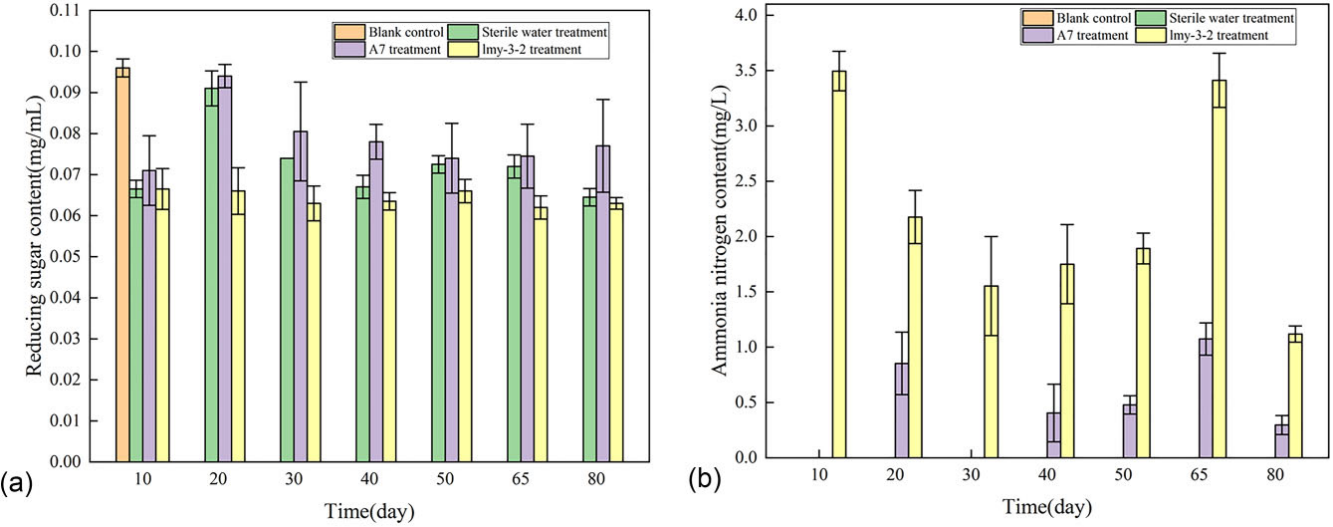
Determination of reducing sugar content (A) and ammonia nitrogen content (B) in potted soil of different treatment groups after 80 days of husk fermentation

The soil reducing sugar content and ammonia nitrogen content after 80 days of fermentation of rice husks treated with composite microbial agents were measured, and the results are shown in Fig. 6A, after soaking the rice husks in bacterial solution and burying them in soil for fermentation, the reducing sugar content in the soil of the experimental group was lower than the original sugar content of 0.096 mg/mL measured in untreated (no husks) soil, and the maximum value was 0.094 mg/mL on the 20th day of chaff fermentation, and then decreased slowly, and the change range was large during the seedling planting period. No ammonia nitrogen content was detected in the untreated soil and sterile water treatment group after chaff fermentation, and there was no change in the ammonia nitrogen content with time. However, the ammonia nitrogen content of potted plants in the compound agent A7 group and nitrogen-fixing monobacterium lmy-3-2 treatment group changed, and the ammonia nitrogen content of A7 group changed with time, and the ammonia nitrogen content was only 1.073 mg/L on the 65th day, as shown in Fig. 6B. The significant changes in nutrient composition in soil under different treatment groups and at different times indicate the vigorous life activities of soil microbial communities, including the degradation of cellulose by cellulose degrading bacteria, the absorption and release of NH4^+^ by nitrogen fixing bacteria, and the utilization of reducing sugars by microbial communities.

### SEM Analysis Results of Surface Microstructure of Chaff

**Fig. 7.**
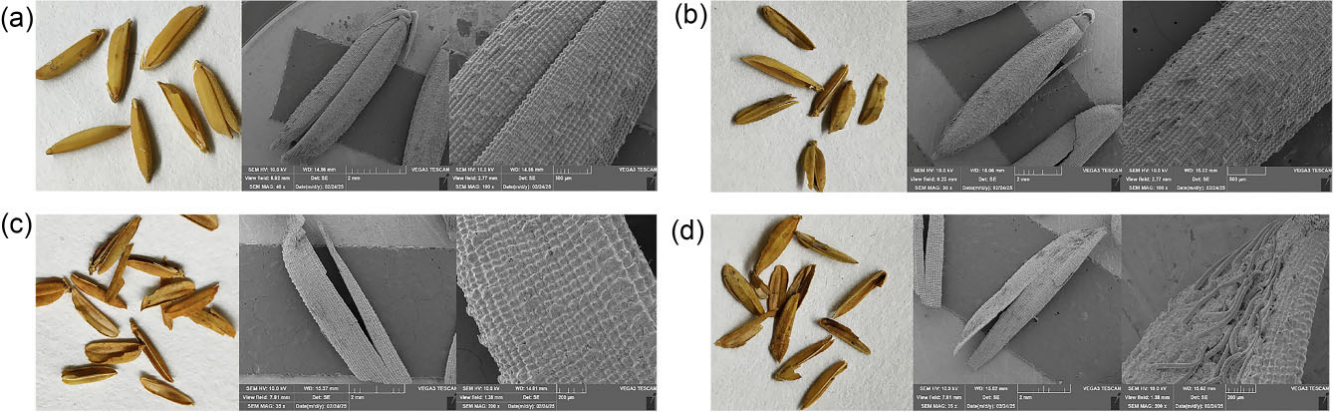
Surface scanning electron microscopy images of rice husks treated with different microbial agents after 80 days of fermentation in soil. A: CK (without any treatment); B: Sterile water treatment; C: Treatment with nitrogen fixing bacteria LMY3-2; D: Compound bacterial agent A7 treatment; The first image is a normal shot, and the last two are enlarged electron microscope scanning images from different fields of view.

In this experiment, the rice husk and the completely untreated rice husk after 80 days of soil burial fermentation were analyzed by SEM, the results are shown in Fig. 7. The surface of the untreated husk is clean under normal shooting, and the structure is neat under SEM, the surface of the husk is inlaid with neat needle-like hairs, and a clear waxy layer can be clearly observed (Fig. 7A). After the treated husk is degraded under soil cover, the surface color of the husk becomes darker under normal shooting. The husk treated by Sterile water treatment (Fig. 7B) and nitrogen-fixing bacteria (Fig. 7C) has the smallest difference from the untreated husk, only a small amount of needle-like hairs on the surface are broken at the base, and pores and gaps appear. The husk treated by A7 (Fig. 7D) has the most serious surface damage, almost all needle-like hairs fall off, some cellular structures are broken and disappear, and a large number of longitudinal thick veins are exposed inside the husk.

## Results of Buckwheat Seedling Pot Planting Experiment

**Fig. 8.**
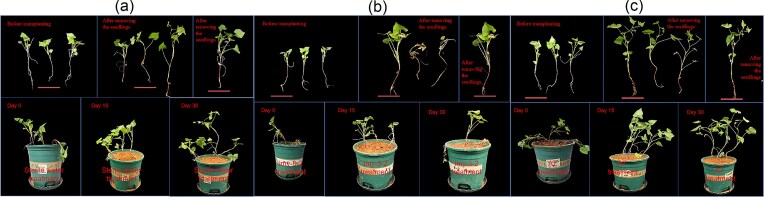
Comparison chart of growth of buckwheat seedlings treated with different microbial agents. A: Sterile water treatment; B: 1my-3-2 single bacterial treatment; C: Compound bacterial agent A7 treatment; The rice husks treated with different bacterial agents were buried in the soil of the potted plants for fermentation, three seedlings were planted in each pot, the top three pictures of each puzzle are the seedling tiling before transplanting, the seedling removal after 30 days of planting, and the seedling single plant tiling shooting after 30 days of planting; the red line marked the seedlings as the same seedling; The following three images were taken from left to right on the 0th, 15th, and 30th day of seedling transplantation to potted plants.

The buckwheat seedlings were transplanted into potted plants on the 20th day of rice husk fermentation, and after 30 days of growth, the buckwheat seedlings went through the flowering and fruiting periods, and were transplanted out of the pot 50 days after the husk fermentation. As shown in Fig. 8, a comparison was made between the growth rate, growth status, and survival rate of seedlings after seedling removal in the early and late stages of transplanting. Compared with the Sterile water treatment group and the nitrogen-fixing single bacteria treatment group, the growth condition of the potted seedlings treated with compound agent A7 was good, and the plant height changed greatly before and after, and the growth effect of the potted seedlings treated with compound agent A7 (cmk-7 + lmy-3-2) was the best compared with the growth status of the initial seedling height and growth process. As shown in Table 3, the plant height growth of the compound agent A7 treatment group was increased by 96.5% and 193.9% compared with the Sterile water treatment and nitrogen-fixing single bacteria treatment, and the seedling growth condition was good. This result indicates that the rice husks in potted plants treated with compound bacterial agent A7 can effectively promote crop growth compared to the sterile water treatment group and the nitrogen-fixing single bacterium treatment group.

**Table 3.** Plant Height Changes of Potted Buckwheat Seedlings

Treament	Plant height bfore transplanting/cm	Plant height after removing seedlings/cm	Growth/cm
Sterile water	12.16	20.25	8.09
lmy-3-2	11.93	17.34	5.41
A7	12.97	28.87	15.9

### Metagenomic Sequencing Results of Soil Microorganisms

#### Effect of compound microbial agent A7 on soil bacterial community diversity

**Fig. 9.**
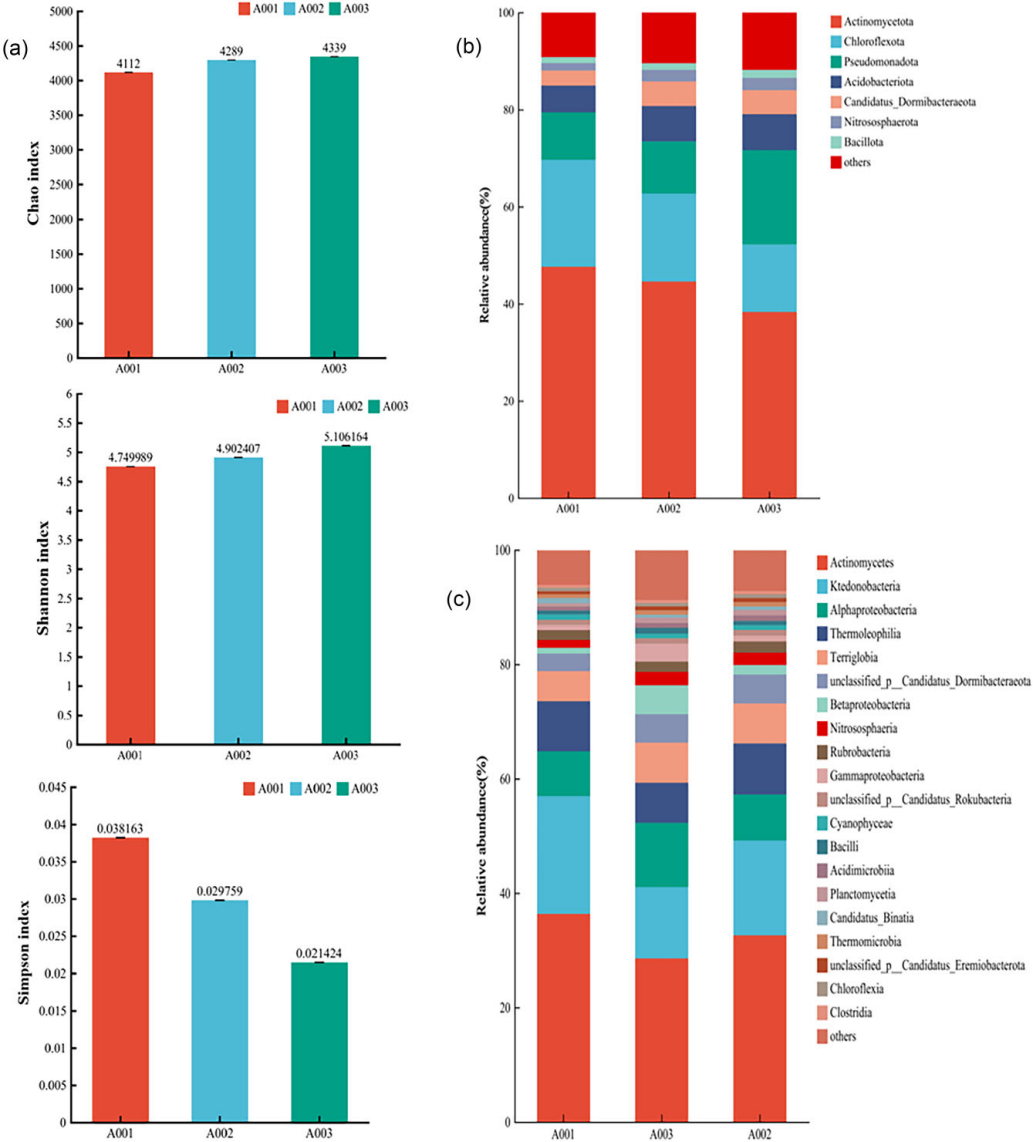
Soil bacterial community α-diversity and community composition at different levels. A: Soil bacterial genus level α-diversity index; B: Bacterial community composition at phylum level; C: Composition of bacterial communities at the genus level. A001 is the untreament (No husk fermentation) group; A002 is a sterile water treatment group; A003 is the A7 treatment group of compound microbial agent.

The results of α-diversity analysis (Fig. 9A) of soil bacterial genus level after inoculation with nitrogen-fixing compound bacteria group A7 showed that Chao index and Shannon index of nitrogen-fixing compound bacteria group A7(A003) were significantly higher than those of untreated group (No husk fermentation) (A001) and sterile water treatment group (A002). The Simpson index of A7(A003) was significantly lower than that of untreated group (A001) and the sterile water treatment group (A002), which indicated that the A7 treatment group had the richest species number, evenness and diversity of soil communities, so it can be preliminarily judged that the inoculation of nitrogen fixing composite microbial agent A7 changed the α-diversity and richness of soil microorganisms.

#### Analysis of abundance changes of A7 members of compound microbiota in soil

The relative results of species abundance at the phylum level (Fig. 9B) showed that the dominant phylum *Actinobacteriota* was mainly present in the untreated group, and the relative abundance decreased in the husk fermentation treatment group. The relative abundance of *Pseudomonadota* was significantly higher than that of the no treatment group and the blank treatment group after the treatment of compound agent A7, and the relative abundance of *Acidobacteriota* and *Candidatus_Dormibacteraeot* was also significantly higher than that of the untreated group. The relative results of species abundance at the genus level (Fig. 9C) showed that the dominant species such as *Ktedonobacter, Conexibacter, Trebonia*, and *Pseudonocardi* mainly gathered in untreated soil, and the abundance of these species decreased after husk fermentation, especially after the husk is treated with compound agents A7, the decrease was significant. On the contrary, the relative abundance of *unclassified_p__Candidatus_Dormibacteraeota* and *Sphingomonas* increased significantly after the use of compound agent A7, and the change of the relative abundance of different bacterial genera led to the change of the whole soil microbial community structure.

#### Abundance changes and analysis of growth-promoting genes in soil

**Fig. 10.**
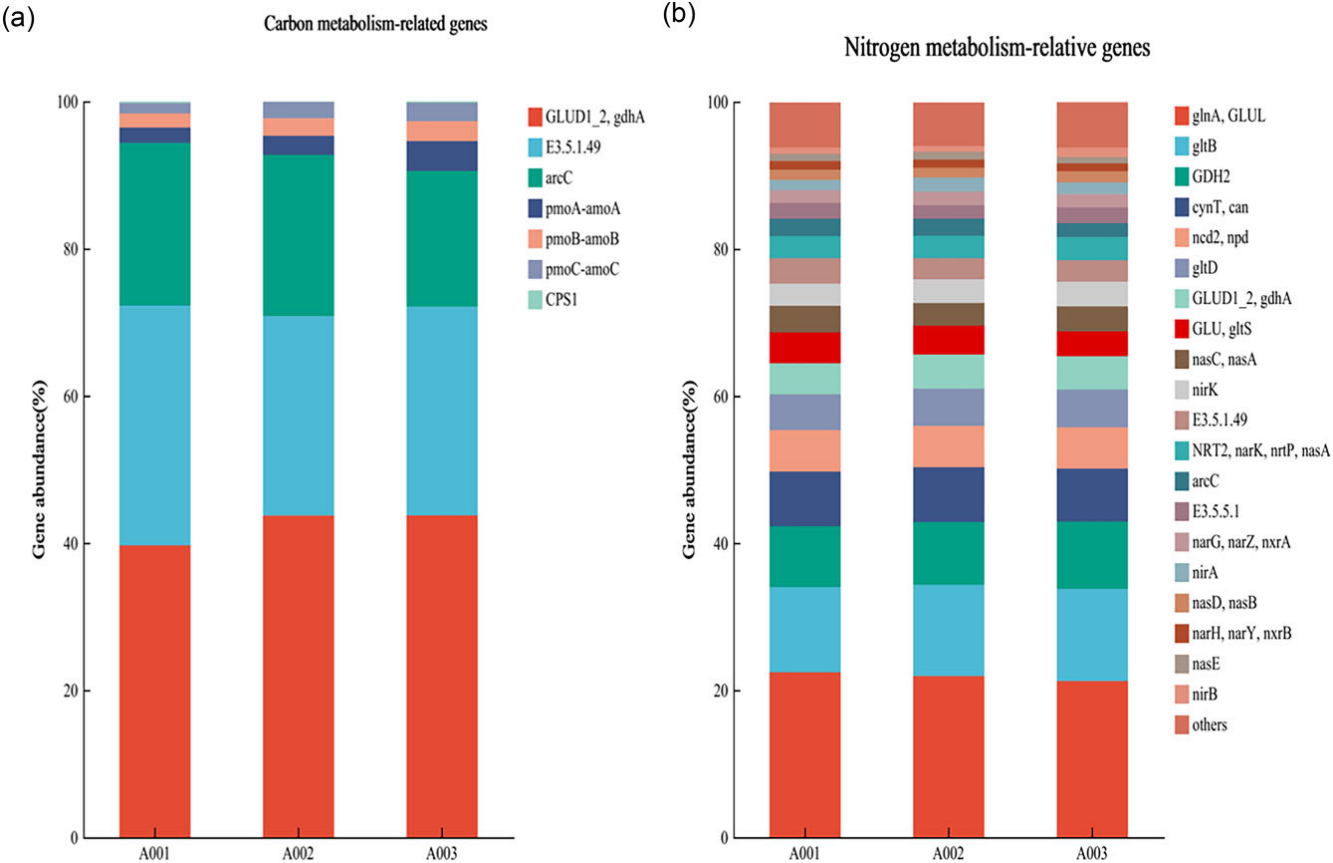
Changes in the abundance of genes related to carbon metabolism (A) and nitrogen metabolism (B) in soil communities. A001 is the untreated (No husk fermentation) group; A002 is a sterile water treatment group; A003 is the A7 treatment group of compound microbial agent.

Based on KEGG database, the related functional genes in metagenomic data are annotated, and carbon metabolism-related genes (Table 4) and nitrogen metabolism-related genes are annotated respectively (Table 5). As shown in Fig. 10, among the genes related to carbon metabolism in soil (Fig. 10A), the abundance of genes GLUD1_2, gdhA, pmoC-amoC, and CPS1 increased significantly in the treatment group with compound microbial agent A7(A003), indicating that the number of functional genes related to carbon metabolism in soil after treatment with compound microbial agent A7 increased, which can improve the carbon conversion efficiency in soil. There are a large number of genes related to nitrogen metabolism in soil (Fig. 10B). Genes such as glnA, GLUL, gltB, GDH2, gltD, GLUD1_2, and gdhA are responsible for encoding amino acid enzymes that participate in the metabolism of amino acids. nasC, nasA, nirK, NRT2, narK, nrtP, nasA, narG, narZ, nxrA, nirA, nasD, nasB, narH, narY, nxrB, nasE, nirB, these are genes involved in the reduction of nitrate and nitrite, with genes cynT, can, and genes ncd2, npd involved in the metabolism of cyanate and urea, respectively. Among the three treatment groups, the abundance of genes such as glnA, GLUL, gltB, GDH2 is higher. After inoculation with compound agent A7, the abundance of genes related to nitrogen metabolism such as gltB, GDH2, gltD, GLUD1_2, gdhA increased in A7(A003) treatment group, it shows that the nitrogen transformation efficiency of soil can be improved by increasing the abundance of nitrogen metabolism after inoculation with compound flora A7. The abundance of genes related to carbon metabolism and nitrogen metabolism reflects the ability of microorganisms to utilize organic carbon and nitrogen, including amino acid synthesis, carbon dioxide fixation and nitrogen assimilation, nitrogen fixation, nitrate reduction, etc., which are essential for the growth and metabolic activities of microorganisms, and also have a direct impact on the acquisition of carbon and nitrogen nutrients by plants through roots.

**Table 4.** Functions of Carbon Metabolism-Related Genes

Gene	Function	References
GLUD1_2, gdhA	Encodes glutamate dehydrogenase, which is involved in the oxidation-reduction reactions of glutamate.	Park & Horton (2019)
E3.5.1.49	Decomposes formamide into ammonia and carbon dioxide.	Campbell et al. (2020)
arcC	Encodes the gene that produces carbamate kinase, which is involved in the conversion of arginine to ornithine	van der Meulen et al. (2019)
pmoA-amoA	Encodes a methyl group in the key enzymeAMO/pMMO, which promotes the decomposition and conversion of organic nitrogen	Deng et al. (2015), He et al. (2021)
pmoB-amoB	Encodes a methyl group in the key enzymeAMO/pMMO, which promotes the decomposition and conversion of organic nitrogen	Lawton et al. (2014)
pmoC-amoC	Encodes a methyl group in the key enzymeAMO/pMMO, which promotes the decomposition and conversion of organic nitrogen	Ahlgren et al. (2019), Ye et al. (2024)
CPS1	Envolved in the fixation of carbon dioxide and encodes a key enzyme (Carbamoyl-Phosphate Synthase 1) in the Calvin cycle.	Matone et al. (2016)

**Table 5.** Functions of Nitrogen Metabolism-Related Genes

Gene	Function	References
glnA, GLUL	Encodes the most critical enzyme for nitrogen assimilation (glutamine synthetase)	Zhang et al. (2020)
gltB	Housekeeping gene involved in nitrogen metabolism, encoding a large subunit of glutamate synthase	Sorty et al. (2025)
GDH2	Encodes glutamate dehydrogenase	Silao et al. (2020)
cynT, can	Encodes carbonic anhydrase enzyme, which catalyzes the production of carbonic acid from carbon dioxide and water	Ramanan et al. (2013)
ncd2, npd	Encodes nitrate monooxygenase	Torres-Guzman et al. (2021)
gltD	Encodes a small subunit of glutamate synthase	Beckers et al. (2001)
GLUD1_2, gdhA	Encodes glutamate dehydrogenase, which is involved in the oxidation-reduction reactions of glutamate.	Park & Horton (2019)
GLU,gltS	Encodes a glutamate transporter	Wang et al. (2023)
nasC, nasA	Genes required for the assimilation of nitrate to nitrite	Nakano et al. (1995)
nirK	Encodes an enzyme that reduces nitrite to nitrogen or nitrous oxide	Zhao & Zhao (2018)
E3.5.1.49	Decomposes formamide into ammonia and carbon dioxide.	Campbell et al. (2020)
NRT2, narK, nrtP, nasA	Encodes a nitrate-to-nitrite transporter	Fukuda et al. (2015), You et al. (2022)
arcC	Encodes the gene that produces carbamate kinase, which is involved in the conversion of arginine to ornithine	van der Meulen et al. (2019)
E.3.5.5.1	Encodes a nitrile hydrolase enzyme that converts nitrile compounds directly into the corresponding carboxylic acids and ammonia	Malaka & Akkaya (2024)
narG, narZ, nxrA	Genes encoding nitrite oxidoreductase and denitrification genes	Maza-Márquez et al. (2022)
nirA	The encoded enzyme reduces nitrate to nitrite	Gazitúa et al. (2021)
nasD, nasB	The subunit encoding nitrite reductase is involved in the bacterial transport and reduction of nitrate and nitrite.	Li et al. (2024)
narH, narY, nxrB	Genes encoding nitrite oxidoreductase and denitrification genes	Maza-Márquez et al. (2022)
nasE	Encodes subunits of nitrite reductase	Li et al. (2024)
nirB	Encodes nitrite reductase	Yılmaz et al. (2022)

## Discussion and Conclusion

In this paper, a strain of *Chromobacterium violaceum* cmk-7 with cellulose degradation ability was screened by carboxymethyl cellulose-Congo red staining method, and the cellulase activity of the strain was determined by quantitative detection of CMCase enzyme activity and FPase enzyme activity, The CMCase and FPase enzyme activities of cmk-7 increased with time, indicating that this chromobacter has the potential to produce cellulase. Therefore, in this study, the fermentation conditions of the strains were explored through single factor experiments. Subsequently, according to the results of the optimization of the single factor test, the three-factor three-level experiment was designed by the response surface method, and the optimal growth conditions of the bacteria were determined to be 1500 µL of inoculation amount, 10 mL of liquid volume and pH 6.0, then, under optimized conditions, the strain was cultured for validation experiments, and the OD_600_ of the strain increased by 2.43 times compared to before optimization. Obviously, optimizing the fermentation conditions of strains through the response surface is an effective means to increase the concentration of bacteria. For example, Wang et al. conducted a series of single factor experiments on three screened cellulase-producing strains: A24, A49, and A61 to determine the best production conditions of CMCase, and further optimized the production conditions of CMCase of A49 by response surface method. According to the predicted conditions, the results of three parallel experiments showed that A49 always produced an average activity of 15.63 U/mL, which was very consistent with the predicted value of 16.41 U/mL, which proved the effectiveness of A49 in optimizing the production conditions of CMCase (Wang et al., 2024).

To date, the main microorganisms found to be able to degrade cellulose are fungi, bacteria, and actinomycetes. Zhu et al. found that inoculation with *Gloeophyllum trabeum* during the composting process could alter the fungal community and significantly increase the activities of various enzymes and the degradation rate of lignocellulose (Zhu et al., 2021). Li et al. studied and determined the optimal enzyme production conditions for the low-temperature cellulose-degrading bacterium Streptomyces azureus T23-B, which significantly improved the degradation capacity of lignocellulose (Li et al., 2021). However, the enzyme system secreted by a single strain is limited and the degradation efficiency of husk is low, and the composite strains constructed by multiple single strains can significantly improve the enzyme activity through the coordination between strains, thereby degrading cellulose more efficiently and converting it into carbon compounds such as monosaccharides that can be used. For example, the compound agent of wax-degrading bacteria constructed by Wang can now improve the degradation rate of straw, and the relative degradation rate of lignin and cellulose-degrading bacteria can reach 30.4%. Through the observation of surface microstructure, it is found that wax-degrading bacteria have a strong destructive effect on the leaf surface, mainly destroying the wax layer (Wang et al., 2021). While microbial degradation of chaff will consume a large amount of nitrogen, so the return of chaff to the field will cause an imbalance in soil C/N ratio and reduce crop yield (Ma et al., 2018). Nitrogen-fixing microorganisms can absorb nitrogen from the air for their own growth and reproduction, and can also secrete excess nitrogen into the soil to provide nitrogen nutrients needed for the growth and reproduction of other organisms (Timofeeva et al., 2023), and studies have shown that the content of phosphorus, potassium, and nitrogen in soils treated with nitrogen-fixing complex microbial agents increases compared with soils applied with a single strain (Cao et al., 2011; Nie et al., 2017). By analyzing the interaction between soil microbial community and soil environmental factors, Liu et al. found that the contents of salt, NH_4_^+^-N and NO_3_-N in the soil planted with Panax quinquefolium increased significantly, while soil pH, C/N ratio, alkaline phosphatase, and cellulase activities decreased significantly, bacterial diversity decreased, and fungal diversity and richness increased. Pearson correlation analysis showed that the significant changes of microbial community were significantly related to the changes of soil pH, soil salt and nitrogen content, alkaline phosphatase and cellulase activity (Liu et al., 2021).

This experiment prepared a composite microbial agent consisting of cellulose degrading bacteria and nitrogen fixing bacteria to ferment rice husks, and conducted pot and seedling experiments, as revealed by SEM, the surface structure of the rice husk was markedly loosened and damaged when compared to the sterile-treated control group. The experimental findings demonstrated that the constructed compound agent A7 exerted a pronounced degrading effect on the rice husk (Wang et al., 2021)_._ Metagenome sequencing analysis of potting soil at the beginning and end of the experiment showed that the composition of the soil microbial community was changed by the compound microbial agent, and the changes in the relative abundance of different bacterial genera and the enrichment of some carbon metabolism and nitrogen metabolism-related genes changed the soil microbial community structure and soil environment after inoculation with the compound microbial agent A7. The compound microbial agent A7 constructed in the experiment is a combination of cellulose degrading bacteria and nitrogen-fixing bacteria. The remarkable changes of nutrient components in the soil indicate that the microbial community in the soil has vigorous life activities (including cellulose degradation and NH_4_^+^ absorption and release), which effectively promotes the degradation of chaff and the growth of buckwheat seedlings.

To sum up, the study of compound microbial inoculum can not only lay a foundation for the study of crop waste degradation, but also develop biological products or agricultural inputs containing compound microbial flora, which is of great significance for promoting crop production.

## Data Availability

Not applicable.
